# Energy – a scoping review for the Nordic Nutrition Recommendations 2023 project

**DOI:** 10.29219/fnr.v67.10233

**Published:** 2023-11-14

**Authors:** Lieselotte Cloetens, Lars Ellegård

**Affiliations:** 1 Division of Pure and Applied Biochemistry, Lund University, Lund, Sweden; 2 Department of Internal Medicine and Clinical Nutrition, Sahlgrenska Academy, University of Gothenburg, Gothenburg, Sweden

**Keywords:** energy, energy balance, metabolic rate, reference values, energy requirements, Nordic, Baltic

## Abstract

We need energy intake to provide energy and nutrients to our cells. The amount of daily energy intake should aim for energy balance, which results in good health. Under- or overconsumption of total daily energy over a longer period leads to increased risk of diseases. In this scoping review, the components of daily energy requirement are defined. Several methods to estimate energy requirements and the amount of total daily energy intake (kJ) related to health are also discussed. Reference values for energy intake in children, adults and pregnant and postpartum women, and older adults are evaluated.

Results show that it is challenging to set reference values for energy intake since existing methods are not accurate and precise, and there are several factors that influence the estimated amount of energy. Energy requirement is increased during growth as in childhood, pregnancy and lactation. We conclude that more research in this area is needed, and that new high-quality studies in both Nordic and Baltic countries are needed to obtain new recommendation numbers for energy intake.

## Popular scientific summary

- Intake of energy is required for all body functions, growth and physical activity.
- Maintaining energy balance supports good health and body weight stability.
- Daily energy requirements are mainly estimated by doubly labelled water measurements or the factorial method.
- Reference values for all life stages in the Nordic and Baltic population are provided

Humans need energy for physical activity and for the regulation of all biochemical processes that maintain body structures and functions. Energy is provided by the diet through the intake of carbohydrates, lipids and proteins (and alcohol). The extent of daily energy requirement is dependent on several factors. In this scoping review, daily energy requirement is defined, and the aim is to describe the reference values for daily energy intakes for children, adolescents, adults, pregnant and lactating women, and older adults.

## Methods

This review follows the protocol developed within the Nordic Nutrition Recommendations 2023 (NNR2023) project (Box 1) (1).

The literature search contained the following terms: (((‘energy’[Title] AND ‘systematic review’ [Publication Type] AND (‘2011’[Date-Publication]: ‘3000’[Date-Publication]) AND Humans [Filter]). The search was conducted on 22.6.2021 using PubMed. The search yielded 220 articles, and only three systematic reviews (2–4) have been selected and included in this scoping review.

We also identified relevant literature, using PubMed, for this scoping review via ‘snowballing’/citation chasing. Furthermore, several reports from the World Health Organization (WHO) and Scientific Advisory Committee on Nutrition (SACN), which have been found via Google search, have been included as references in this scoping review.

*Box 1.* General information about the review according to the guidelines of NNR2023.

- This paper is one of many scoping reviews commissioned as part of the NNR2023 project (1)
- The papers are included in the extended NNR2023 report, but, for transparency, these scoping reviews are also published in Food & Nutrition Research
- The scoping reviews have been peer reviewed by independent experts in the research field according to the standard procedures of the journal
- The scoping reviews have also been subjected to public consultations (see report to be published by the NNR2023 project)
- The NNR2023 committee has served as the editorial board
- Whilst these papers are a main fundament, the NNR2023 committee has the sole responsibility for setting dietary reference values in the NNR2023 project

Some of the references in this scoping review are rather old, and energy requirements for the different groups in the NNR should be re-evaluated in the future. Large high-quality studies in the Nordic and Baltic countries are needed to obtain new reference values for energy requirement.

## Components of daily energy expenditure

### Definitions of energy requirement

The basic principle behind the formulation of energy requirement reference values is *energy balance*, i.e., the physiological state in which daily energy intake equals energy expenditure, and both body weight and energy content (defined by body composition) are constant. For some people, especially those who are over- or underweight, the recommended energy intake might be lower, or higher, than energy expenditure for a prescribed time period, but long-term energy balance is the ultimate goal even in treatment of malnutrition.

NNR defines the energy requirement in adults as the energy intake needed to cover energy expenditure in individuals with body weight, body composition and physical activity compatible with good health. In addition, the energy requirement is affected by several factors, including age, level of physical activity and endocrine changes. Children require an increased energy level for growth. A higher energy requirement per kg body weight is also seen in pregnant women for deposition of tissues, and during lactation for milk production (5)*.*

Body energy stores are very large (at least 30 times the daily energy expenditure), and therefore, there is no need for energy intake and energy expenditure to be equal over short periods of around 1 to 4 days (6).

The daily energy expenditure has three different components:

- Basal (or resting) energy expenditure (BEE or REE)
- Diet-induced thermogenesis (DIT)
- Energy expenditure caused by physical activity

Energy expenditure is measured in kJ (1,000 kJ = 1 MJ) per time unit (usually MJ/d).

On average, daily energy expenditure is higher in men than in women, but the difference disappears after adjustment for the difference in body size and body composition between the sexes.

Very cold or hot environments, genetic differences, hormonal status (e.g. concentrations of thyroid and growth hormones), sympathetic nerve activity, psychological state, pharmacological agents and several diseases have been shown to increase or decrease energy expenditure, mainly by affecting REE (7, 8).

## Basal (resting) energy expenditure

BEE, or basal metabolic rate (BMR), is defined as the energy expenditure of an individual at physical and mental rest in a thermo-neutral environment and in a fasted state. REE is measured under less rigorous conditions than BEE and is considered, therefore, to be approximately 5% higher than BEE. The mean energy expenditure is slightly lower during sleep than during waking hours (7). Therefore, sleeping energy expenditure (SEE) is about 10% lower than BEE. Despite small systematic differences, SEE, BEE and REE are very strongly interrelated, and they are often used interchangeably.

In individuals with approximately equal physical activity levels, daily energy expenditure is strongly related to body weight, and particularly to fat-free mass (FFM; FFM = body weight – fat mass) (9, 10). FFM consists of skeletal muscle and organ tissue. Fat mass (FM) also shows a positive correlation with energy expenditure. However, the increase in energy expenditure per unit FM is much smaller than for unit FFM (9). Hence, the inter-individual variations in FFM explain much more of the REE compared to variations in FM. When expressed per kg, the metabolic rate in internal organs is much higher than in skeletal muscle. In adults, 70–80% of BEE is derived from organs that comprise only 5% of the total body weight (9). Thus, there is an association between total FFM and REE, such that when FFM (and hence muscle mass) is low, the slope of BEE against FFM is lower than when FFM (and muscle mass) is high (8). Thus, when the organs make up a higher proportion of the FFM, increases in skeletal muscle mass have less influence on REE.

The inter-individual variation at a given FFM is about 2.1 MJ per day, and this indicates the possible magnitude of the difference in REE between two individuals with similar FFM. Variations in genetic and metabolic profile, body composition, hormone concentrations, energy balance and physical fitness have been found to explain the variation in REE after adjustment for FFM (7, 8, 10–12).

## Diet-induced thermogenesis

DIT, or diet-induced energy expenditure, is defined as an increase above REE in energy expenditure after food intake divided by the energy content of the food ingested (13). The postprandial rise in energy expenditure lasts for several hours, but about 90% of DIT is observed within 4 h of the meal. DIT is assumed to be 10% of the daily energy expenditure in individuals in energy balance who consume a mixed diet with a food quotient corresponding to 0.85 (10, 14, 15). The DIT of fat is only about 5% of its energy content, whilst the DIT of protein is approximately 20%. The DIT of carbohydrate is around 10% of its energy content. It might be 20% in rare occasions if glucose is directly converted to fat (de novo lipogenesis). However, this process requires a huge excess of energy from carbohydrates, which rarely occurs in healthy individuals consuming diets typical for the Nordic and Baltic countries (16).

## Physical activity

*Physical activity* (at work or leisure time) is defined as any bodily movement produced by skeletal muscle that results in energy expenditure (17). *Exercise* is a subcategory of physical activity and is a voluntary, deliberate physical activity performed because of anticipated positive effects on physical, psychological and/or social well-being.

The daily physical activity level (PAL) is defined as total energy expenditure divided by REE (or BEE) (Table 1). This way of quantifying physical activity assumes that the variation in daily energy expenditure is based on physical activity and body size.

**Table 1:** Physical activity measurements

Parameters related to physical activity	Physical activity level (PAL)	Metabolic equivalent task (MET)
Definition	Total energy expenditure/REE	Energy expenditure during a physical activity/REE
Time frame of measurement	Mean daily measurement (24 h)	Instant measurement (min or h)
Range of values	1.1–2.4	1.0–15.0

REE = resting energy expenditure.

The metabolic equivalent of task (MET; MET = energy expenditure during an activity divided by REE) is a measure of instant physical activity level, and PAL is the daily average of the METs weighted by the time each task (Table 8) was performed (Table 1) (18, 19). The inter-individual variation in PAL is much more restricted than for MET, which can range, for example, from 1.2 when sitting to as high as 15 for riding a bicycle at a speed of 30 km/h.

Daily physical activity (and physical activity-induced energy expenditure) can be divided into occupational and leisure activities, which both vary in grades of intensity. Inactivity refers to a state where energy expenditure is close to REE, and this usually includes sitting or lying down whilst awake. The associations amongst physical activity, sedentary lifestyle and health are described in detail in a scoping review on physical activity (20).

## Energy balance and health

### Body mass index

Body mass index (BMI) is defined as body weight (kg) divided by the square of the height (m^2^). BMI has a U- or J-shaped association with total mortality and morbidity (21–23). In general, the BMI compatible with the lowest mortality (and morbidity) in adult Caucasians is approximately 22–23 kg/m^2^. According to the WHO definition (21), the normal (or recommended) BMI is between 18.5 and 24.9 kg/m^2^ (Table 2). The term pre-obese describes a slightly elevated BMI (25–29.9 kg/m^2^), and a BMI of 30 kg/m^2^ or more is defined as obesity. Overweight is defined as all subjects having a BMI of ≥25.0 kg/m^2^.

**Table 2:** Body mass index; definitions of underweight, overweight and obesity; and health risks for adults 20–64 years of age (21, 25)

Body mass index (kg/m^2^)	Definition	Morbidity and mortality
<18.5	Underweight	Slightly increased
18.5–24.9	Normal weight	Low
>25.0	Overweight	
25.0–29.9	Pre-obese	Slightly increased
30.0–34.9	Grade I obesity	Increased
35.0–39.9	Grade II obesity	Much increased
≥40.0	Grade III obesity	Very much increased

In obesity, the amount (in kg or as a percentage of body weight) or anatomical distribution (subcutaneous/visceral or abdominal/truncal) of body fat leads to an increased risk for adverse health effects, particularly type 2 diabetes, cardiovascular diseases, musculo-skeletal disorders and cancer. Regardless of whether the amount of body fat or the distribution of body fat is used, it is not possible to determine a single point separating normal and healthy body weight from obesity. Moreover, health risks increase with increasing severity of obesity (21, 22, 24, 25).

The categories in Table 2 are, in principle, applicable in all Nordic and Baltic countries. However, it should be kept in mind that BMI might represent different levels of fatness and body fat distribution depending on age, sex, ethnicity, athletic training and race. For instance, the healthy BMI range might be higher for Inuits (26) and lower for individuals of Asian descent (27). Therefore, BMI on the individual level should be used with great caution. Other simple measures, such as waist circumference (see the *Abdominal obesity* section), might help to assess obesity-related health risks.

In a meta-analysis (28), the sensitivity of BMI for detecting high adiposity was 0.50 (95% confidence interval (CI): 0.43–0.57), and its specificity was 0.90 (CI: 0.86–0.94). These data indicate that using BMI leads to both type I errors (obesity is detected even when it is not true) and type II errors (true obesity is not detected), and that type I errors seem to be more common. Okorodudu et al. (28) compared BMI against measures of body fat from body composition analyses and showed that BMI is more prone to underestimate than to overestimate body fatness. In other words, many individuals with a BMI just below a cut-off limit (e.g. 25 or 30) should have been classified as overweight or obese, respectively. Despite a common belief, it is less typical that BMI overestimates fatness (although it certainly does, for example, in well-trained athletes and bodybuilders) (29, 30).

Obesity in children and adolescents can be defined using BMI, but the cut-off points differ from those presented in Table 2. Cole et al. (31) have published international age- and sex-specific BMI cut-off points for overweight (85th percentile) and obesity (95th percentile) for children and adolescents between 2 and 18 years. Furthermore, ISO-BMI, which is a BMI adjusted for age and sex, has been developed by Obesity Task Force, making it possible to convert the child’s BMI into an adult equivalent to diagnose overweight and obesity (32). Many countries also use specific age-adjusted growth charts (weight-to-height for a given age) to assess overweight and obesity.

Studies have found that ageing is associated with changes in body composition along with a loss of muscle mass and a gain of body fat (33, 34). These changes imply that optimal BMI might be different in older people compared to younger people. Several studies have found the BMI associated with the lowest age-adjusted mortality to be higher in elderly people when compared to recommendations for younger subjects (34, 35). The national board of health and welfare of Sweden suggests that an optimal BMI for elderly is between 23 and 29 kg/m^2^ (36). Unfortunately, the current available data are inadequate to make any precise international recommendations for optimal BMI amongst the elderly.

The prevalence of adult obesity in the Nordic and Baltic countries is shown in Table 3. These data were obtained from WHO European Regional Obesity report (2022) (25) and show that roughly half of the adult population in Nordic and Baltic countries is either overweight or obese. Because individuals tend to underreport their body weight, the actual prevalence of overweight and obesity is likely to be somewhat higher than shown in Table 3. Compared to the prevalence of obesity reported in NNR 2012, this disease has become more common in Sweden (37) than the other and Baltic and Nordic countries (25).

**Table 3:** Estimated prevalence (%) of adult overweight and obesity (-age standardised) in the Nordic and Baltic countries (from WHO 2022) (25)

Countries	Overweight, including obesity			Obesity		
	Both sexes	Women	Men	Both sexes	Women	Men
Denmark	55.4	47.3	63.6	19.7	17.0	22.3
Finland	57.9	50.0	65.6	22.2	20.6	23.7
Iceland	59.1	50.5	67.5	21.9	19.4	24.2
Norway	58.3	51.4	65.0	23.1	22.5	23.6
Sweden	56.4	48.5	64.2	20.6	18.1	23.1
Estonia	55.8	51.9	59.6	21.2	21.8	20.3
Latvia	57.8	54.9	60.9	23.6	25.1	21.6
Lithaunania	59.6	56.5	62.6	26.3	27.8	24.2

Very recently, the National Academies of Sciences, Engineering, and Medicine (NASEM) has published a report about reference dietary energy intake (38). The prevalence of overweight (defined as BMI between 25.0 and 30.0 kg/m^2^ and thus excluding obesity) in the US is 35.4 ± 1.2% and 27.8 ± 0.7% in adult men and women, respectively. The prevalence of obesity (defined as BMI ≥ 30.0 kg/m^2^) in US adults is ranging from 32.5 to 45.4%, which is higher than in the Nordic and Baltic countries. Notably, less than half of the US adult population (28.8%) has a normal body weight.

In this report, the daily reference intake population is now defined as the general population, including those with overweight, obesity and chronic diseases, and thus no longer defined as ‘generally healthy’ population.

### Abdominal obesity

Abdominal fat distribution is an indicator of intra-abdominal fat mass and can also be used as an indicator of obesity (39). Table 4 presents cut-off points for waist circumference (40) as also suggested by the National Institutes of Health (41) and the WHO (21). Intra-abdominal fat mass, or abdominal fat distribution, can be even more strongly associated with metabolic disturbances than the total amount of body fat. The cut-off points are probably higher for elderly subjects (35, 42, 43), but BMI values are interpreted without any age adjustments in all adults older than 18 years.

**Table 4:** Waist circumference (cm) and the risk of metabolic complications in adults (18–64 years)

Risk level	Women	Men
Low	≤79	≤93
Increased	80–87	94–101
High	≥88	≥102

### Obesity, weight stability and health

Obesity, and to a smaller extent overweight, is associated with an increased incidence of several diseases (24). This meta-analysis found statistically significant associations between obesity and overweight and the incidence of type 2 diabetes, several types of cancers (breast, endometrial, colorectal and kidney), cardiovascular diseases, asthma, gallbladder disease, osteoarthritis and chronic back pain. The strongest association was found between obesity and type 2 diabetes.

According to epidemiological studies, stable weight is related to the lowest total mortality, and weight gain is clearly related to increased mortality (44). Many epidemiological studies indicate that weight loss is also associated with increased mortality (44–48). However, these data should be interpreted with caution because of difficulties in separating voluntary and involuntary (due to pre-existing disease) weight reduction. Moreover, epidemiological studies do not separate different techniques or rates (and/or extent) of weight reduction or composition of lost body weight (49). Nevertheless, even a modest (5–10%) weight reduction in high-risk individuals significantly improves health (21). Weight cycling (weight reduction and then increasing to previous weight) might have adverse effects on mortality and morbidity (50, 51), but the results are contradictory (52–54).

### Determinants of obesity and weight control

Weight gain is caused by a positive energy balance. Several retrospective and prospective population-based studies have evaluated factors related to obesity or weight gain.

The effect of dietary macronutrients and food consumption as determinants of long-term weight change has been reviewed by Fogelholm et al. (55). This review found probable evidence that high intake of dietary fibre and nuts predicted less weight gain, and that high intake of meat predicted more weight gain (55). Suggestive evidence was found for a protective role against increased weight for whole grains, cereal fibre, full-fat dairy products and high scores on an index describing a prudent dietary pattern. Likewise, there was suggestive evidence for both fibre and fruit intakes as a protection against increases in waist circumference. Suggestive evidence was found for high intake of refined grains, sweets and desserts in predicting weight gain, and for refined (white) bread and a high energy density diet in predicting increases in waist circumference. The results of this literature search suggested that the proportion of macronutrients in the diet was not important in predicting changes in weight or waist circumference. A meta-analysis by Wycherley et al. showed however that the consumption of a high protein diet has more modest benefits on weight control compared to a standard protein diet (2). In contrast, prospective cohort studies have shown that increased intake of fibre-rich foods and dairy products and a reduction in refined grains, meat and sugar-rich foods and drinks are associated with a less weight gain.

In a meta-analysis, Te Morenga et al. (56) concluded that the intake of free sugars or sugar-sweetened beverages is a determinant of increasing body weight. These results give additional support for restricting sugar intake as a means to prevent obesity. In another meta-analysis, Chen et al. (57) did not find evidence that the intake of dairy products prevented weight gain. These results are somewhat in contrast to the review by Fogelholm et al. (55). The discrepancies in the results might be related to different selection of studies; Chen et al. (57) scrutinised randomised trials, but Fogelholm et al. (55) examined cohort studies. A post-hoc analysis was performed to investigate potential evidence for association between grouped exposure variables and grouped outcome variables (BMI and waist circumference not separated) (55).

**Fig. 1:**
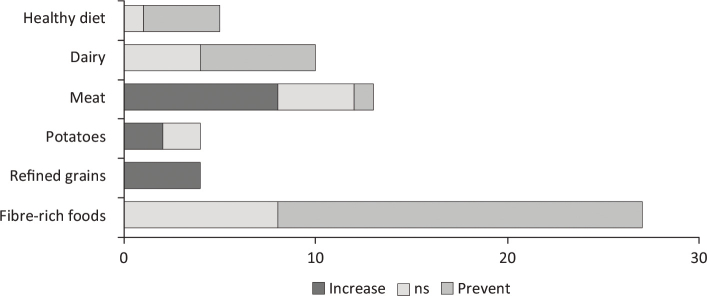
Evidence for association between grouped exposure variables and grouped outcome variables (BMI and waist circumference not separated) (55). BMI = body mass index.

These analyses suggest that a healthy diet in general (assessed using indices that describe the healthiness of dietary patterns) and fibre-rich foods are clearly associated with less weight gain (Fig. 1). Dairy products are only to some extent associated with reduced weight gain. In contrast, refined grains, and sugar-rich foods and drinks are associated with more weight gain. Furthermore, the consumption of meat, in general, shows an increase in weight gain. However, it is important to mention that the intake of lean meat with a higher protein content and less saturated fatty acids, as part of a healthy diet, could contribute to weight loss and weight maintenance (58).

The level of physical activity is another important lifestyle factor and determinant of obesity and weight control. Low levels of physical activity are positively associated with obesity and age-related weight gain, whereas high levels of physical activity are associated with less weight being regained after weight reduction (59). The intensity and duration of physical activity might also affect the extent of weight control (60). However, most of the above findings are observational and retrospective, and the studies are still inconclusive as to whether physical activity can be regarded as a single predictor of weight control. Spontaneous physical activity corresponds to small, involuntary muscle movements, such as fidgeting, and might be related to weight control (61), but the data to support this are limited. Obesity is also associated with education level and socioeconomic status. The general trend is that higher socioeconomic strata infer lower prevalence of obesity compared with lower socioeconomic strata (37, 62, 63).

## Methods to estimate energy requirements

There are two main approaches to estimate total energy requirements. The first approach is the doubly labelled water (DLW) technique (64–66). DLW in the gold standard to measure energy expenditure in free-living conditions (67). This method is based upon the oral intake of water labelled with stable isotopes (^2^H and ^18^O). The isotopes are gradually eliminated from the body, through water and ^18^O through water and CO_2_. The difference between the elimination rates of ^2^H and ^18^O, measured by isotope ratio mass spectroscopy, is related to CO_2_ production and, therefore, to energy expenditure. This estimation of total energy expenditure is quite accurate, provided that the experimental and analytical conditions are appropriate. In theory, a large number of DLW measurements could be used as the basis to predict total energy expenditure by deriving equations that describe how total energy expenditure varies as a function of, for example, age, sex and various anthropometric measures such as weight and body fat. There are several data sets with energy expenditures for a total of several hundred individuals assessed by DLW (68, 69) and pooled analyses (70). However, the populations in these studies were selected, and the representativeness of these data cannot be guaranteed.

The second main approach to assess energy expenditure is the factorial method in which total energy expenditure is calculated from the resting (or basal) energy expenditure (REE) and a factor indicating PAL. DLW is more accurate in assessing individuals, but the factorial method provides more opportunity to generalise the results. Therefore, estimates of average energy requirements in the previous and current NNR were determined using the factorial method.

Because of technical constraints on REE measurements, determinations of energy requirements are usually based on predicted REE. Table 5 shows prediction equations for REE as given by Henry (71).

**Table 5:** Equations for calculating the average resting energy expenditure (REE, MJ/d) based on either body weight (W, kg) or a combination of weight and height (H, m) (71)

Age Year	REE	REE
	MJ/d based on weight	MJ/d based on weight and height
Girls		
<3	0.246 W – 0.0965	0.127 W + 2.94 H – 1.20
3–10	0.0842 W + 2.12	0.0666 W + 0.878 H + 1.46
11–18	0.0465 W + 3.18	0.0393 W + 1.04 H + 1.93
Women		
19–30	0.0546 W + 2.33	0.0433 W + 2.57 H – 1.180
31–60	0.0407 W + 2.90	0.0342 W + 2.10 H – 0.0486
61–70	0.0429 W + 2.39	0.0356 W + 1.76 H + 0.0448
>70	0.0417 W + 2.41	0.0356 W + 1.76 H + 0.0448
Boys		
<3	0.255 W – 0.141	0.118 W + 3.59 H – 1.55
3–10	0.0937 W + 2.15	0.0632 W + 1.31 H +1.28
11–18	0.0769 W + 2.43	0.0651 W + 1.11 H + 1.25
Men		
19–30	0.0669 W + 2.28	0.0600 W + 1.31 H + 0.473
31–60	0.0592 W + 2.48	0.0476 W + 2.26 H – 0.574
61–70	0.0543 W + 2.37	0.0478 W 0 + 2.26 H – 1.070
>70	0.0573 W + 2.01	0.0478 W 0 + 2.26 H – 1.070

REE = resting energy expenditure.

Recently, a systematic review on the estimation of energy expenditure in overweight and obese adults, including 21 different studies, concluded that there is to date no single prediction equation, providing both accurate and precise estimations of REE (72). However, the Mifflin equations are recommended, as precision is considered more important than accuracy in clinical practice (73).

The NASEM report mentions that the same total energy expenditure equations can be used for normal and overweight/obese BMI categories (38). They also conclude that the interaction between BMI and resting energy expenditure is limited. Furthermore, they noticed that equations to measure total energy expenditure are marginally better when using height and body composition compared to height and body weight.

Very recently, new standard equations for future DLW studies have been proposed by Speakman et al. (74). These equations might simplify the calculations, but they have not been generally accepted yet (74).

## Reference values for energy requirements in children and adolescents

Part of the energy intake of children and adolescents is used for growth, and their energy requirement per kg body weight is, therefore, higher than for adults. During the first 4 months of life, approximately 27% of the energy intake is used for growth. At the end of the first year of life, this amount decreases to approximately 5%; at age 1–3 years, it decreases to approximately 3%; and in older children, this value is less than 2% (75).

Reference values for energy requirements of children and adolescents should be based on their REE, their energy expenditure in response to physical activity and their energy requirements for growth. These values should be consistent with the attainment and maintenance of long-term good health, including recommended levels of physical activity (76).

### Age 1–12 months

The estimated energy requirement for infants is based upon the approach of FAO/WHO/UNU (5) where daily energy expenditure is calculated using DLW-derived equations (77) (Table 6).

**Table 6:** Estimated average daily energy requirements (per kg body weight) for children 1–12 months assuming a mixture of breastfeeding and complementary foods (77)

Age months	Average daily energy requirements kJ/kg body weight	
	Boys	Girls
1	486	469
3	411	404
6	339	342
12	337	333

Some studies have shown that breast-fed infants have a lower energy intake than formula-fed infants (78–80), especially infants breast-fed more than 7 months, and that this results in less body weight gain from 6 to 10 months than in infants breast-fed for a shorter period (81, 82). The effect of the infant’s food source on energy requirements was found to persist throughout the second year of life in one of the studies used as a basis for the estimated energy requirement (75). This was primarily because of a higher REE in formula-fed than in breast-fed infants (75) although varying digestibility might also play a role (83). However, the differences between feeding groups in terms of energy expenditure never exceeded 20 kJ/kg, and the current NNR gives a single energy requirement that is valid for both breast-fed and formula-fed infants.

### Estimated average reference values for children and adolescents

The estimated daily energy requirements according to age for children and adolescents (Table 7) are based on the factorial method. Thus, REE is first estimated using the equations of Henry taking into account weight and height (71), and daily energy expenditure is then calculated by multiplying REE by an appropriate PAL. In Table 7, the values for body weight related to age in the group aged 2–5 years are based on the mean of the reference values from Denmark (84), Estonia (85), Finland (86), Norway (87) and Sweden (88).

**Table 7:** Estimated daily energy requirements (MJ/d) for children and adolescents (from 2 to 17 years) in the Nordic and Baltic countries using the Henry equations for REE (71) and the physical activity levels from SACN (77)

Age (y)	Boys		Girls		Estimated energy requirement MJ/d at different physical activity levels^a^					
					Boys			Girls		
	Weight (kg)	BMR (MJ/d)	Weight (kg)	BMR (MJ/d)	Low	Average	High	Low	Average	High
2	13.2	3.19	12.4	2.94	4.30	4.43	4.56	3.97	4.08	4.20
3	15.2	3.51	14.6	3.27	4.74	4.88	5.02	4.42	4.55	4.68
4	17.4	3.,75	16.8	3.49	5.32	5.98	6.34	4.95	5.48	5.89
5	19.3	3.96	19.0	3.70	5.63	6.22	6.69	5.25	5.80	6.25
6	21.9	4.23	21.6	3.94	6.01	6.64	7.15	5.60	6.19	6.66
7	24.6	4.48	24.0	4.15	6.37	7.04	7.58	5.90	6.52	7.02
8	27.2	4.72	26.7	4.38	6.71	7.42	7.98	6.23	6.88	7.41
9	30.1	4.98	29.8	4.64	7.07	7.82	8.41	6.59	7.28	7.84
10	33.3	5.25	33.5	4.94	8.72	9.08	9.71	8.20	8.54	9.14
11	36.9	5.29	37.7	4.95	8.79	9.16	9.79	8.22	8.57	9.16
12	41.4	5.65	42.9	5.22	9.38	9.78	10.45	8.67	9.03	9.66
13	47.0	6.09	48.0	5.48	10.12	10.54	11.27	9.09	9.48	10.13
14	53.2	6.57	52.3	5.68	10.91	11.37	12.16	9.44	9.83	10.52
15	59.4	7.04	55.3	5.82	11.69	12.18	13.03	9.67	10.07	10.77
16	64.2	7.39	57.5	5.92	12.27	12.79	13.68	9.83	10.25	10.96
17	67.8	7.65	58.8	5.98	12.71	13.24	14.16	9.93	10.35	11.07

REE = resting energy expenditure; SACN = Scientific Advisory Committee on Nutrition; BMR = basal metabolic rate.

a Physical activity levels (low, average and high) by age group. 1–3 y: 1.35, 1.39 and 1.43; 4–9 y: 1.42, 1.57 and 1.69; 10–18 y: 1.66, 1.73 and 1.85.

Values for growth at school age show increasing weight-to-height ratios and an increased prevalence of overweight (25). This means that using current weight data would base the recommendations on an increasing prevalence of excess body weight. Therefore, weight values for 6–17 year olds are calculated from measured height (84–88) and BMI according to WHO (2007) (89). Body weight reference values have increased compared to NNR 2012, especially from 8 years and older. For boys, the mean increase is 3.4% with the highest increase at the age of 14 years (7.5%). The mean increase for girls is 2.8%, with the highest increase at 11 years (58%).

Children of the same age vary widely in body weight, particularly in the age groups where only a small fraction of the children have started puberty. The body weight of children of the same age and sex can differ by a factor of two. Therefore, the estimated energy requirement in a certain age group, as illustrated in Table 7, must be used with caution. It is also important to mention that the use of the equation at age boundaries (3–4 years and 9–10 years) might be ambiguous. Moreover, the calculated energy requirement for overweight children (>2SD weight-to-height ratio) is too high when based on body weight because such children have a comparatively high body fat content, and the energy requirement is primarily determined by the size of the FFM. Therefore, it is recommended that the energy requirement in overweight children should be based on the weight one SD above normal weight for height or on the weight corresponding to the cut-off value of overweight according to the International Obesity Task Force (21).

PAL values in the NNR2023 are based on a systematic review of the DLW studies that were carried out for the SACN (77) recommendations. The analysis showed no significant differences between the sexes but did show an increased PAL with age. We have used the first quartile (25th percentile) value as a cut-off for low vs. average activity, and the third quartile (75th percentile) as the cut-off for average vs. high activity (Table 7).

If we compare data calculated according to the NNR2023 with the recommended equations from NASEM (38), the energy expenditure values are similar with a tendency for a slightly higher REE calculated by NASEM. For example, a 2-year boy (98 cm and 15.5 kg) has a REE of 5.28 MJ/d according to NNR2023 and 5.36 according to NASEM calculations. REE for an adolescent male (15 years, 170 cm, 66 kg, and active PAL) is 12.86 MJ/d and 13.35 for NNR and NASEM calculations, respectively. PAL levels defined by NASEM are divided into four levels (inactive, low active, active and very active), and the range of PAL values of each level is depending on age of the child.

## Reference values for energy requirements in adults

The reference values for energy requirements in adults are based on estimates of REE and PAL. Energy requirement is equivalent to the product of REE and PAL. BMR can be calculated from the prediction equations that are presented in Table 5, and the PAL values (Table 8) are estimated generalisations (average values) based on studies using DLW. By using more detailed information on daily physical activity (time spent in different activities) and the respective MET values (19), PAL can be approximated for an individual as the daily time-weighted average MET value (Tables 8 and 9). For instance, in Table 9, an active day is assumed to consist of 8 h rest (mostly sleep), 10 h very light activity (mostly sitting, sometimes standing) and 2 h light activity (e.g. slow walking, cooking, etc.). In addition, the day consists of 1 h moderate activity (e.g. brisk walking) and 1 h vigorous activity (e.g. playing football). To calculate PAL, the MET values of different activity levels are multiplied by the time spent in the corresponding activity divided by 24. Daily energy expenditure is calculated by multiplying PAL by the REE.

**Table 8:** Physical activity level expressed as multiples of the resting energy expenditure according to different levels of occupational and leisure activity

Description of physical activity levels	PAL
Bed-bound or chair-bound (not wheelchair)	1.1–1.2
Seated work with no option of moving around and little or no leisure activity	1.3–1.5
Seated work with some requirement to move around, and with some leisure activity	1.6–1.7
Work including both standing and moving around (e.g. housework and shop assistant) OR seated work with some requirement to move around with regular, almost daily, leisure activity	1.8–1.9
Very strenuous work or daily competitive athletic training	2.0–2.4

Source: Modified from Black et al. (70).

Note 1: Moderate leisure physical activity (e.g. brisk walking): 0.025 PAL unit increase for each hour per week.

Note 2: Strenuous leisure physical activity (e.g. running and competitive football): 0.05 PAL unit increase for each hour per week.

**Table 9:** Two examples of how to estimate daily physical activity levels from data on different physical activity levels

	Very inactive day		Active day	
Intensity of activity (MET)	Time, h	MET × h	Time, h	MET × h
Rest (1.0)	10	10	8	8
Very light (1.5)	12	18	10	15
Light (2.0)	2	4	4	8
Moderate (5.0)	0	0	1	5
Strenuous (10.0)	0	0	1	10
Total	24	32	24	46
PAL	1.33		1.92	

**Explanation**. The time spent in different activities is multiplied by the respective metabolic equivalent value (MET value). To obtain the daily physical activity level (PAL), the sum of daily MET × h is divided by 24. Hence, PAL is the weighted average of daily MET × h. Daily energy expenditure is calculated by multiplying PAL by the resting (or basal) energy expenditure.

An average PAL for adults in Nordic and Baltic countries is assumed to be around 1.6, which is compatible with sedentary work and some physical activity (68, 69). A totally sedentary lifestyle (PAL 1.4–1.5) is associated with health risks that might be equal to the risk associated with marked obesity (BMI 30–35) or regular smoking. These health risks are offset by approximately 3–4 h per week of moderate physical activity or 2 h per week of more strenuous leisure-time physical activity (90), which would mean an increase of only 0.1 PAL units. However, it is likely that a PAL of roughly 1.8 would be more optimal for overall health. This level was close to the 75th percentile in the large data sets of Tooze et al. (68) and Moshfegh et al. (69). This PAL is approximately the same as that observed in moderately active prepubertal children (91). Strenuous athletic training can increase energy requirements to PAL 2.0–2.5 and in extreme cases even up to 4.0 (92, 93). However, it is rare for physical exercise to increase energy requirements by more than 20% compared to energy expenditure during normal daily living. PAL 1.4 is used as the level indicating physical inactivity, and this level is close to the 15th percentile in larger population samples (68, 69).

Table 10 shows reference weights based on population data in Denmark (94), Estonia (95), Finland (96), Iceland (97), Latvia (98), Norway (99) and Sweden (100). Because of the high prevalence of overweight and obesity, population weights cannot be used directly to estimate reference weights because then the reference energy needs would support the maintenance of overweight and obesity. Therefore, the reference weight needs to be adjusted to a theoretical situation in which all individuals are at normal weight. The reference weight was calculated by using population-based data on height to estimate an age-adjusted weight corresponding to BMI 23. This arbitrary BMI was used to indicate healthy weight. The precise mean point within the WHO normal body weight range (BMI 18.5 to 24.9) would have been BMI 21.7. Because the actual mean BMIs of the populations in all Nordic and Baltic countries are clearly higher, BMI 23 was chosen as more realistic but still within the normal BMI range.

**Table 10:** Reference weights (kg) from Nordic and Baltic countries calculated as the weight for height corresponding to BMI 23

Age groups for men and women	Denmark^a^	Estonia^b^	Finland^c^	Iceland^d^	Latvia^e^	Norway^f^	Sweden^g,h^	Mean
Men, age in years								
18–24	74.9	75.0	75.9	75.5	73.9	76.5	74.5	75.2
25–50	75.0	74.6	73.9	75.5	74.5	75.4	74.9	74.8
51–70	72.1	71.6	71.7	75.4	71.2	74.7	74.5	73.0
>70	69.1	68.9	68.4	73.2	68.9	73.6	72.1	70.6
Women, age in years								
18–24	64.5	64.8	61.9	63.3	64.5	65.1	64.9	64.2
25–50	64.8	63.9	62.8	64.6	63.9	64.8	63.8	64.1
51–70	62.2	60.6	60.8	64.5	61.6	64.3	63.7	62.5
>70	60.1	58.8	57.4	62.5	60.6	62.9	61.9	60.6

BMI = body mass index.

Data sources:

a (94),

b (95),

c (96),

d (97),

e (98),

f (99),

g (100).

h The age groups for Sweden: 18–30 years, 31–44 years, 45–64 years and >65 years.

Table 11 shows the average estimates of daily energy requirements for men and women with respect to age, different activity levels and reference weight (Table 10). The values in Table 10 are estimations, assuming that all individuals have BMI 23. It should be noted that these estimations have a large standard error due to imprecision in both estimation of REE and of PAL. Therefore, the results should be used only for estimations on the group level. In particular, the data for the oldest age group in Tables 10 and 11 should be used with special caution. Due to the age-related weight changes amongst healthy elderly individuals, 0.5–1.0 kg should be subtracted from the average weights in Table 10 for every 5 years above the age of 75.

**Table 11:** Reference energy requirements (MJ/d) in adults based on Nordic and Baltic reference weights (Table 10) and different physical activity levels

Age, years	Reference weight, kg^a^	REE, MJ/d^b^	Sedentary PAL^c^ 1.4	Average PAL 1.6	Active PAL 1.8
Men					
18–24	75.2	7.4	10.4	11.8	13.2
25–50	74.8	7.1	9.9	11.3	12.7
51–70	73.0	6.4	9.0	10.3	11.6
>70	70.6	6.3	8.8	10.1	11.3
Women					
18–24	64.2	5.9	8.3	9.4	10.6
25–50	64.1	5.7	8.0	9.0	10.2
51–70	62.5	5.2	7.2	8.3	9.3
>70	60.6	5.1	7.1	8.2	9.2

a Reference weight corresponds to BMI 23.

b REE = Resting Energy Expenditure, estimated from the equations of Henry (71). The REE for 18–24 year olds was calculated with the equation 19–30 y, the REE for 25–50 year olds was calculated with the equation 31–60 y, and the REE for 51–70 year olds was calculated with the equation 61–70 years.

c PAL = Physical Activity Level.

The mean daily energy intake in adults living in the Nordic and Baltic countries has also been given in Lemming and Pitsi (101). Values for men ranged from 8.7 (Estonia) to 11.2MJ (Denmark) and for women from 6.5 (Estonia) to 8.4 MJ (Denmark). These data are generated from dietary surveys and have no specific age ranges and levels of physical activity considered.

Reference values for energy requirements are based on assumptions regarding weight stability, normal (healthy) weight and energy balance. However, these assumptions are not always valid. For instance, a negative energy balance is needed for the treatment of obesity. If the energy intake is 2.1 MJ/d below the requirement for energy balance, the estimated weight reduction during the first month is approximately 500 g/week. This rate of weight loss is often recommended although a larger negative energy balance (up to 4.2 MJ/d) leading to a weight loss of 1,000 g/week still seems to be compatible with a healthy weight reduction (13, 102). The long-term estimation (several months to years) of weight loss due to a fixed reduction in energy intake is much more complicated (103). The reason for this is that energy expenditure decreases with weight loss. Hence, with increasing weight reduction, the energy deficit decreases (same intake but less expenditure). Therefore, the 500 g/week weight loss for each 2.1 MJ (500 kcal) reduction in energy intake cannot be used for anything other than predicting initial weight reduction.

The energy requirement for an individual with weight and physical activity different from the values presented in Tables 10 and 11 can be calculated as follows. First, the RMR is estimated using the appropriate equation in Table 5. PAL is then estimated either from Table 8 or using the calculation shown in Table 9. Finally, the energy requirement is calculated as RMR × PAL. It should be noted, however, that RMR as well as PAL tends to be imprecise, and it is indeed possible to misjudge the daily energy requirement by at least 2 MJ.

Compared to NASEM calculations (38), REE values measured by NNR’s suggested formula are slightly lower. For example, a woman (22 years, 165 cm, 63 kg and low active PAL) has an REE of 8.10 and 9.52 MJ/d, calculated by the methods of NNR and NASEM, respectively.

## Energy requirement during pregnancy

The requirement for energy during pregnancy is based on estimates of weight gain during gestation and the composition of that gain in terms of fat and protein. A review and meta-analysis, which is rated as moderately confident according to Amstar2-NNR, concluded that the mean weight gain during pregnancy was 12.0 (2.8) kg (1, 3). In a study including 95 Swedish pregnant women, the median weight gain was very similar, i.e., 12.1 (10.0–15.3) kg (104). This value is, however, lower than average values for weight gain in pregnancy in the other Nordic countries, which ranges between 14.0 and 16.8 kg (104–108, 109), and NASEM (110) has extended the recommendation of weight gain in pregnancy by taking varying pre-pregnancy weight into consideration.

Pregnant women are in an anabolic dynamic state throughout gestation, and this creates additional needs for energy. Forsum and Löf described the partitioning of energy metabolism in the pregnant versus the non-pregnant state (111). According to Butte and King (112), ‘The energy requirement of a pregnant woman is the level of energy intake from food that will balance her energy expenditure when the woman has a body size and composition and level of physical activity consistent with good health’. The energy requirement of pregnant women includes the energy needs associated with the deposition of tissues consistent with optimal pregnancy outcome (112). The energy cost in pregnancy is due to the foetus, placenta and amniotic fluid as well as the weight gain of the uterus and breasts and increased volumes of blood, extracellular water and adipose tissue (113).

The mean energy expenditure during pregnancy has been studied by Savard et al. (4). This systematic review included 32 studies that have measured total energy expenditure and/or REE in women with singleton pregnancies. Most of the studies (75%) were performed in Europe and North America. According to the Amstar2-NNR, the review is graded as critically low. Results of this review are shown in Table 12. However, confounding variables, such as pre-pregnancy weight and BMI, dietary intake and physical activity, are not considered and might result in inconsistency.

**Table 12:** Median increase in REE and TEE during each trimester of the pregnancy (4)

Type of trimester during pregnancy	Median increase in REE	Median increase in TEE
1st trimester	5.3% (72 kcal)	6.2% (144 kcal)
2nd trimester	9.9% (153 kcal)	7.1% (170 kcal)
3rd trimester	18.0% (252 kcal)	12.0% (290 kcal)

REE = Resting Energy Expenditure.

An additional aspect that should be considered is the potential decrease in energy needs due to a decrease in physical activity during pregnancy. This is a complicated issue where definite answers cannot be provided. Studies have shown that Swedish pregnant women do (114) or do not (115) ‘save energy’ by such a decrease. Thus, as stated by Prentice et al. (116), it cannot be assumed that a high proportion of the energy costs of pregnancy are normally or automatically met by reductions in physical activity.

There is large variation amongst women regarding the amount of weight gained during pregnancy. Weight gain during pregnancy amongst women in the Nordic countries is, on average, between 14.0 and 16.8 kg (105–109). Positive associations between this gain and the health of both baby and mother have been observed. However, a very large weight gain is a health risk both for mother and child, especially amongst women who were overweight or obese prior to pregnancy (e.g. an increased risk for breast cancer in the mother, spontaneous abortion, gestational diabetes and gestational hypertension) (105, 117). If weight gain during pregnancy is too small, the risk for a low-birth-weight baby is increased because weight gain in pregnancy is positively correlated to infant size at birth (105). Low birth weight increases the risk for health complications in early life and has been found to be related to increased risks of adult diseases such as coronary heart disease, hypertension and type 2 diabetes (105, 118–120). Within Europe, however, the Nordic and Baltic countries have the lowest prevalence of low birth weight (121).

The average birth weight in the Nordic and Baltic countries is high (>3,500 g), and highest in Iceland and the Faeroe Islands, and has been increasing for full-term babies in all these countries in recent years (122). Values on weight gain during pregnancy have been reviewed, and in 2009, the National Academy of Medicine published guidelines with recommended gestational weight gains for women having different BMIs before conception (123). These are the values now recommended by NNR for Nordic and Baltic women (Table 13).

**Table 13:** Weight gain during pregnancy as recommended by the Institute of Medicine (123)

BMI (kg/m^2^) before conception	Recommended weight gain (kg)
<18.5 (underweight)	12.5–18.0
18.5–24.9 (normal weight)	11.5–16.0
25.0–29.9 (overweight)	7.0–11.5
>30.0 (obese)	5.0–9.0

BMI = Body mass index.

In recent years, the importance of foetal nutrition has attracted a significant amount of interest. Studies in humans as well as in experimental animals suggest that the supply of energy and nutrients during this very first part of life is related to health later in life. Furthermore, studies have shown that the nutritional situation of the woman before conception is also important, and, as indicated earlier, in the US, the recommended weight gain during pregnancy varies according to the pre-pregnancy BMI of the woman. In fact, recommendations, also from the US (123), emphasise that ‘all women should start pregnancy with a healthy weight’, i.e., with a BMI between 18.5 and 24.9 kg/m^2^. A systematic literature review (124) shows that insufficient data are available regarding health outcomes of intended weight loss as a result of dieting prior to conception. It is conceivable that such weight loss might be associated with harmful effects, for example impaired iron and folate status during subsequent pregnancies and a risk for developing eating disorders. NNR2023 suggests that nutrition therapy can be applied for a moderate weight reduction amongst women with obesity prior to conception.

Overweight and obesity are common amongst Scandinavian and Baltic women of reproductive age, and this is a serious concern because the pre-pregnancy BMI is a strong predictor of many adverse outcomes of pregnancy (25, 123). Therefore, it is important that every effort is made to avoid overweight and obesity in women of reproductive age. However, although overweight and obesity are presently the most common nutritional problems in Nordic and Baltic women, it should be emphasised that low BMI and insufficient weight gain do occur in some women and are associated with increased health risks for their offspring.

## Energy requirement during lactation

The additional energy requirement during lactation is based on estimates of the energy costs for milk production and an estimate of the amount of energy mobilised from the body’s energy stores. During pregnancy, there is a physiological retention of body fat that, to some extent, can be mobilised postpartum. Thus, the energy needs during lactation are dependent on the nutritional status of the mother during pregnancy. According to Butte and King (112), ‘The energy requirement of a lactating woman is the level of energy intake from food that will balance her energy expenditure when the woman has a body size and composition and a breast milk production which is consistent with good health for herself and her child and that will allow for desirable physical activity’.

According to international recommendations (5, 112), energy requirements during lactation for women in developed countries are based on an average milk production of 749 g every 24 h. For partial lactation, the breast milk production is assumed to be 492 g every 24 h. Table 14 shows the energy cost of lactation for women in developed countries during different time periods postpartum (5, 112). These costs should be added to the energy requirement of the non-pregnant and non-lactating woman, and they can be covered by an increased intake of dietary energy or partly covered by mobilised body fat. This contribution of body fat to the energy costs of lactation has been estimated to be, on average, 0.72 MJ every 24 h during the first 6 months of lactation. However, the variation between individual women is considerable.

**Table 14:** Energy cost of milk production (MJ/24 h) for women in developed countries during exclusive and partial breastfeeding (112)^a^

Months postpartum^b^	0–2	3–5	6–8	9–11	12–23
Exclusive breastfeeding	2.49	2.75	2.81	3.15	–
Partial breastfeeding	2.24	2.40	2.07	1.53	1.57

a These costs can be covered by an increased intake of energy from food or by mobilised body fat (0.72 MJ/24 h on average) during the first 6 months of lactation.

b Scandinavian women are recommended to breastfeed exclusively during the first 6 months postpartum and then breastfeed partially at least until the child is 1 year old.

A large individual variation is certainly also present with respect to the milk production figures given earlier. There are no data showing that lactating women decrease their physical activity to ‘save energy’ for milk production. However, because of a risk for weight gain after pregnancy (125), it is recommended that lactating women increase rather than decrease their amount of physical activity. Recently, recommended level of physical activity for pregnant and postpartum women has been added to the WHO guidelines (126). These guidelines suggest that all pregnant and postpartum women without contraindication should undertake regular physical activity, do at least 150 min of moderate-intensity aerobic physical activity throughout the week for substantial health benefit and incorporate a variety of aerobic and muscle-strengthening activities.

The increased prevalence of overweight and obesity amongst women living in the Nordic and Baltic countries is also a potential problem during lactation because it has been shown that obese and overweight women tend to have a less successful lactation than normal-weight women (127). Furthermore, there are data from Danish women showing that breastfeeding promotes postpartum weight loss (128). However, this effect is rather weak, and it is quite possible to gain weight during lactation if the energy balance is positive, i.e., too much energy from food and/or too little physical activity. A Swedish study showed that dietary advice to overweight and obese lactating women could effectively promote weight loss after pregnancy (129). It is important to stress, however, that breastfeeding is an energy-demanding process, and for many lactating women, a considerably increased energy intake is recommended. A systematic review, rated as critically low according to NNR Amstar2, showed that most of the studies did not show an association between breastfeeding and postpartum weight loss (130).

Because of different study designs, it is difficult to compare results, and more high-quality studies are needed to study the relationship between breastfeeding and postpartum weight loss. Moreover, breastfeeding should be promoted for its health benefits for both mother and child, and not to compensate for excessive weight gain or to promote postpartum weight loss.

The effect of a dietary and physical activity intervention, alone or combined, on weight loss in postpartum women has been studied. We found three reviews, all rated as high or convincing according to Amstar2-NNR, that studied this topic (131–133).

A review by Lim et al. concluded that a combination of a dietary and physical activity intervention is more effective compared to exercise only. Self-monitoring also resulted in greater weight loss compared to women without self-monitoring (131). Another review analysed 12 interventions and concluded that exercise alone did not result in greater weight loss compared to women without exercise (132). However, they observed that a dietary intervention with or without exercise has significantly a greater weight loss than women with usual care. Dodd et al. included 27 studies and concluded that a postpartum dietary and physical activity, alone or combined, intervention resulted in greater weight loss after childbirth compared to women without intervention (133).

From these findings, we conclude that a dietary intervention alone or in combination with exercise has a beneficial effect on weight loss in postpartum women. The effect of increased physical activity without changes in diet on body weight after childbirth seems still unclear. Future research should contain high-quality trials with focus on the optimal duration of the intervention and methodology.

## Energy requirements in older adults

Daily energy expenditure tends to decline with age (134, 135) mainly due to decreased FFM (136, 137) and decreased physical activity (137–139). REE is strongly related to FFM, which consists mainly of muscle and organ mass (137, 140). The decrease in REE is not fully explained by the age-related decrease in FFM (141), and Pannemans et al. (134) found that 80% of the variation in REE in older adults was explained by FFM.

Longitudinal (142–144) and cross-sectional (90, 145, 146) studies have found an age-related decrease in REE, but knowledge about daily energy expenditure in the elderly (>75 years) is limited (90). A Swedish study found that the REE amongst 91–96 year old subjects was not different from the REE amongst subjects between 70 and 80 years (147), whilst a US study found a 27% lower REE in very old individuals compared to 60–74 year olds (148). However, a longitudinal follow-up of the 73 year olds at age 78 showed a decrease in REE as well as TEE but not in active energy expenditure (AEE) (149). The PAL values in the above individuals averaged 1.74 at both ages (73 years and 78 years), indicating a physically active lifestyle for this age group (149). DIT does not seem to be affected by age (146).

A review including 24 studies with measured REE in healthy elderly (mean age 70.6 ± 5.1 years, mean body weight 72.4 ± 6.0 kg and mean BMI 25.6 ± 1.5 kg/m^2^) found the mean of the weight adjusted REE to be about 80 kJ/kg body weight in both males and females, and this value was not significantly different from a group of sick elderly patients (150). The measured PAL obtained from 24 h TEE relative to the REE was 1.66 ± 0.11 amongst the healthy elderly.

Also, for older adults, the REE calculated by the recommended NNR2023 formula is similar with a tendency towards slightly higher values obtained by NASEM recommended formulas (38). For example, a woman of 70 years (157 cm and 70 kg, inactive PAL) has an REE of 7.42 and 7.58 according to NNR and NASEM, respectively.

## Low energy intake

Lowenstein (151) has suggested a reference value of 1,500 kcal/d – corresponding to approximately 6.5 MJ/d – as the minimum daily energy intake necessary for providing an adequate intake of micronutrients from an ordinary diet. In the NNR, *very low energy intake is defined as an energy intake below 6.5 MJ/d*, and an energy intake of 6.5–8 MJ is considered a *low energy intake* with increased risk of an insufficient intake of micronutrients.

A very low energy intake is related to a very low PAL and/or to a low body weight. Low body weight is related to low muscle mass and, therefore, to low energy expenditure. The age-related decrease in energy expenditure might result in a very low energy intake, and such low intakes are also found amongst people on slimming diets and amongst subjects with, for example, eating disorders or food intolerances.

Amongst healthy subjects, very low habitual energy intakes are probably rare – even amongst sedentary elderly subjects, the estimated daily energy requirement is only 7–8 MJ, see Table 11. However, with lower body weight amongst the sedentary elderly, energy intake might become critically low.

The intake of most micronutrients is positively associated with energy intake, and, consequently, habitually low energy intake is associated with low nutrient intake. In dietary surveys, the reporting of energy intake is often biased by a widespread underreporting that is independent of age, and more frequent especially amongst women and people with overweight/obesity. Thus, it is difficult to explore the consequences of low energy intake on nutritional status based on low-energy *reporters*.

Amongst elderly subjects, low reported energy intakes were not associated with biochemical signs of nutritional deficiencies (19, 152). This somewhat surprising result might be explained by underreporting (thus true intakes are higher), or that recommended biochemical levels are already reached at lower intakes than expected. Amongst elderly Europeans (153), it was not possible to establish a level of reported energy intake that ensured an adequate supply of iron, thiamine, riboflavin or pyridoxine. At a reported intake of 8 MJ per day, 13% of men and 16% of women still had an inadequate intake of at least one of these four micronutrients.

## Energy content of foods

### Calculation of energy content

The energy in foods available for metabolism – i.e., the metabolisable energy – is determined by the energy content of the food as assessed in the laboratory by measuring the heat produced when its organic components are fully oxidised. Not all energy in a food item is available to humans, and its energy value must be corrected for losses due to insufficient absorption and, in the case of protein, also for incomplete oxidation and for losses as urea in urine. Accurate calculation of the metabolisable energy content in foods requires knowledge of the foods’ macronutrient content as well as of the digestibility of these macronutrients. Because the energy content and the digestibility of each macronutrient vary between foods, it is convenient to use standardised factors based on the energy content and digestibility of macronutrients representing the composition of an average mixed diet.

Due mainly to historical background and tradition, there are standard factors that differ slightly from each other. In the NNR, the energy content of a mixed diet is calculated based on 17 kJ/g protein and available (glycaemic) carbohydrate and 37 kJ/g fat. Alcohol (ethanol) is considered to yield 29 kJ/g. In kcal, these standard factors are 4 kcal/g protein and carbohydrate, 9 kcal/g fat and 7 kcal/g alcohol. Note that these numbers include some errors caused by rounding off from kilojoules. To transform values between the two systems of units, the following relationships are used: 1 kcal = 4.2 (or 4.184) kJ and 1 kJ = 0.24 (or 0.239) kcal. These standard factors are not intended for calculating the metabolisable energy content in individual food items because the heat of combustion as well as the digestibility varies slightly between macronutrients from different foods. In a mixed diet, however, these variations balance each other, and the standard factors have been shown to be accurate. Specific factors for calculating energy content in individual food items have been presented (154, 155).

The energy content of foods is not fully available to cover human energy requirements. Large differences exist in the amounts of energy available from different macronutrients because their metabolism *per se* requires different amounts of energy. The postprandial rise in energy expenditure is highest for proteins (about 20% of the energy content), lower for carbohydrates (about 10%) and lowest for fat (about 5%) (14, 15). In addition, the absorption of macronutrients varies amongst individuals and is dependent on the specific foods eaten, how they are prepared and intestinal factors (103, 156).

### Carbohydrates and fibre

The values for carbohydrate that are shown in food composition tables are in many cases determined by means of the ‘difference method’ that defines total carbohydrate as the difference between the total dry matter and the sum of protein, fat and ash. These values include digestible mono-, di- and polysaccharides (starch) as well as non-digestible carbohydrates such as lignin and organic acids. The glycaemic or ‘available’ carbohydrates represent total carbohydrates minus dietary fibre and are the sum of the total amounts of sugars and starch.

The heat of combustion of glycaemic carbohydrates is slightly lower for monosaccharides than for disaccharides and even higher for polysaccharides (155). However, these differences can be disregarded in most practical situations. When total carbohydrate is analysed ‘by difference’, available carbohydrate and dietary fibre are considered to contribute with the same amount of metabolisable energy. The energy content will, therefore, be overestimated in diets containing high amounts of dietary fibre if the calculation is based on a carbohydrate content assessed ‘by difference’.

In diets containing up to 30 g fibre per day, standard energy factors can be used without significant consequences for the calculated metabolisable energy content of the diet (156). In fact, dietary fibre contributes only a small amount of such energy because its components are, to some extent, fermented in the colon. End products in this process are short-chain fatty acids that can be absorbed and metabolised, thus contribute to the metabolisable energy of the diet. The magnitude of this contribution depends on the type of fibre, and 8 kJ/g has been suggested as an average value (154, 157–159). In the NNR2023, the energy content of dietary fibre is changed from 0 to 8 kJ/g since this is in line with the suggestions of the Alimentarius Commission and European Nutrition Labelling Directive.

The digestibility of carbohydrates varies from 90% in fruits to approximately 98% in cereals. The digestibility of flour depends on the fractions included, i.e., the digestibility decreases with a higher content of fibre.

### Protein

Protein is not completely oxidised in the body. Therefore, when calculating the metabolisable energy content of protein incomplete digestibility as well as urea losses in the urine must be considered. The digestibility of protein in humans depends on the processing; whole wheat has a digestibility of 86%, refined wheat 96% and wheat gluten 99% (156).

### Fat

The heat of combustion for dietary fat is a function of the fatty acid composition of the triglycerides in the diet and the proportion of other lipids in the diet. On average, the digestibility of dietary fat is considered to be 95% in most foods (154, 155).
